# Potential Defects and Improvements of Equivalent Uniform Dose Prediction Model Based on the Analysis of Radiation-Induced Brain Injury

**DOI:** 10.3389/fonc.2021.743941

**Published:** 2022-01-11

**Authors:** Qing-Hua Du, Jian Li, Yi-Xiu Gan, Hui-Jun Zhu, Hai-Ying Yue, Xiang-De Li, Xue Ou, Qiu-Lu Zhong, Dan-Jing Luo, Yi-Ting Xie, Qian-Fu Liang, Ren-Sheng Wang, Wen-Qi Liu

**Affiliations:** ^1^ Department of Radiation Oncology, Second Affiliated Hospital of Guangxi Medical University, Nanning, China; ^2^ Department of Radiation Oncology, First Affiliated Hospital of Guangxi Medical University, Nanning, China

**Keywords:** equivalent uniform dose, brain injury, predictive ability, volume-effect parameter, nasopharyngeal carcinoma

## Abstract

**Purpose:**

To study the impact of dose distribution on volume-effect parameter and predictive ability of equivalent uniform dose (EUD) model, and to explore the improvements.

**Methods and Materials:**

The brains of 103 nasopharyngeal carcinoma patients treated with IMRT were segmented according to dose distribution (brain and left/right half-brain for similar distributions but different sizes; V*_D_* with different *D* for different distributions). Predictive ability of EUD_V_ *_D_* (EUD of V*_D_*) for radiation-induced brain injury was assessed by receiver operating characteristics curve (ROC) and area under the curve (AUC). The optimal volume-effect parameter *a* of EUD was selected when AUC was maximal (mAUC). Correlations between mAUC, *a* and *D* were analyzed by Pearson correlation analysis. Both mAUC and *a* in brain and half-brain were compared by using paired samples *t*-tests. The optimal D*_V_* and V*_D_* points were selected for a simple comparison.

**Results:**

The mAUC of brain/half-brain EUD was 0.819/0.821 and the optimal *a* value was 21.5/22. When *D* increased, mAUC of EUD_V_ *_D_* increased, while *a* decreased. The mAUC reached the maximum value when *D* was 50–55 Gy, and *a* was always 1 when D ≥55 Gy. The difference of mAUC/*a* between brain and half-brain was not significant. If *a* was in range of 1 to 22, AUC of brain/half-brain EUD_V55 Gy_ (0.857–0.830/0.845–0.830) was always larger than that of brain/half-brain EUD (0.681–0.819/0.691–0.821). The AUCs of optimal dose/volume points were 0.801 (brain D_2.5 cc_), 0.823 (brain V_70 Gy_), 0.818 (half-brain D_1 cc_), and 0.827 (half-brain V_69 Gy_), respectively. Mean dose (equal to EUD_V_ *_D_* with *a* = 1) of high-dose volume (V_50 Gy_–V_60 Gy_) was superior to traditional EUD and dose/volume points.

**Conclusion:**

Volume-effect parameter of EUD is variable and related to dose distribution. EUD with large low-dose volume may not be better than simple dose/volume points. Critical-dose-volume EUD could improve the predictive ability and has an invariant volume-effect parameter. Mean dose may be the case in which critical-dose-volume EUD has the best predictive ability.

## Introduction

Dose/volume parameters are widely used to estimate the probability of normal tissue injury (1–4). However, these parameters are only part of the information contained in the dose volume histogram (DVH) curve. For instance, dose delivered to 1 cc (D_1 cc_) is only a discrete point on DVH. Some normal tissue complication probability (NTCP) models utilize all information of the DVH curve by compressing the entire curve into a single factor (5–7). However, most DVH reduction models are based on estimated complication probability under uniform irradiation (5), which could not be used for non-uniform dose distributions directly. To solve this problem, the equivalent uniform dose (EUD) is often introduced (8–10).

EUD is a simple power-law assumption that may not correspond to the weight changes of all doses. If the doses vary over a wide range, this uncertainty may increase and lead to a decrease in predictive ability—the fitting of power exponent (*a* or 1/*n*) should be more accurate in a small dose range than in a large dose range. Since both volume and dose are factors of EUD formula, the proportions of volume bins in different dose ranges should theoretically influence the volume-effect parameter. To increase the weight of critical dose, the power exponent might be larger in the case that the proportion of inessential low-dose volume becomes larger. In order to identify these potential defects and try to improve them, this study explored the impact of dose distribution on the EUD-based prediction model of radiation-induced brain injury (BI) for nasopharyngeal carcinoma (NPC) patients, taking into account the large enough low-dose volume and typical symmetrical objects including structure, field setup and location of injury.

## Methods and Materials

### Patient Selection and Radiation Therapy

A total of 103 NPC patients treated with intensity-modulated radiotherapy (IMRT) in 31 fractions and concurrent platinum-based chemotherapy were retrospectively reviewed from January 2009 to March 2015 (**Table 1**). All patients were followed every 3 months in the first 2 years and every 6 months during the next 3 years, and then annually thereafter. The median follow-up time was 69.2 months (range, 61.2–120.8 months). Full-course radiation planning was designed and optimized by inverse treatment planning system, 7 (18/103) or 9 (85/103) isocentric fields being set up. The prescribed dose was 68–72 Gy to the planning target volume (PTV) of gross tumor volume (GTV), 60–64 Gy to the PTV of high-risk clinical target volume (CTV), and 50–54 Gy to the PTV of low-risk CTV.

**Table 1:** Basic characteristics for 103 patients.

Characteristics	Injury	Non-injury	P
Gender			0.64
Male	25 (75.8%)	50 (71.4%)	
Female	8 (24.2%)	20 (28.6%)	
Age (median)	45	42.5	0.40
T stage*			<0.01
T1	0 (0%)	0 (0%)	
T2	2 (6.1%)	13 (18.6%)	
T3	8 (24.2%)	40 (57.1%)	
T4	23 (69.7%)	17 (24.3%)	
Brain injury	33		–
Left	11		
Right	13		
Both	9		

*When T stage and dosimetric parameters were analyzed together in multivariate analysis, T stage was removed (P >0.05).

### Toxicity Endpoints

The MRI images were reviewed by two radiologists and a radiation oncologist. Diagnostic criteria for BI were as follows (4): 1) solid lesions with small nodular enhancements on postcontrast T1-weighted sequence, or ring lesions with “Swiss cheese” or “soap bubble” patterns, featuring marginal enhancements and central necrosis; 2) focalized or extensive edema surrounding necrosis, typically presented on T2-weighted images as finger-like areas with hyperintensity; and 3) no evidence of intracranial NPC invasion. Patients who met all the criteria were diagnosed as BI.

### Segmentation for Different Reference Volumes

The brain was contoured in each case as only the pure brain parenchyma was considered, excluding the cavernous sinuses, the brainstem, optic chiasm, optical tract, pituitary gland, mammillary bodies, and Meckel’s caves (11, 12). At the overall level, the dose distribution of left and right half-brain should be similar to that of whole brain (analyzed in results) because the isocentric fields were symmetrical, and the tumors were generally in the middle. In order to obtain reference volumes with similar dose distributions but different sizes, the brain was divided into left half-brain and right half-brain according to brain midline (13). Reference volumes with different dose distributions were obtained directly from DVH (V_0 Gy_ to V_70 Gy_, per 5 Gy).

### Dosimetric Parameters

EUD was calculated with the equation:


$$\text{EUD}={\left(\sum_{i}v_{i}D_{i}^{a}\right)}^{\frac{1}{a}}$$

where *D_i_* is the *i*th dose bin (1 Gy/bin) of a DVH, *v_i_* is the relative volume of that bin and *a* is the volume-effect parameter (8). For comparison purposes, D*_V_* (dose delivered to volume *V*), V*_D_* (volume covered by doses ≥*D*) and EUD_V_ *_D_* (EUD of V*_D_*) were calculated.

### Statistical Analysis

SPSS 19.0 was used for statistical analysis. The V*_D_* proportions (relative volume) of whole-brain and half-brain were compared by using independent sample *t*-tests. Receiver operating characteristic curve (ROC) was used for screening dosimetric parameters to predict BI. The predictive ability was assessed by the area under the curve (AUC). The *a* value in EUD model was adjusted from 1 to 30. The optimal volume-effect parameter *a* of brain/half-brain was selected (or median when there were multiple optimal *a* values) when AUC of EUD model was maximal (mAUC). The correlations between mAUC, *a* and *D* were analyzed by Pearson correlation analysis. The mAUC/*a* in brain and half-brain were compared by using paired sample *t*-tests. The optimal simple dose/volume points were selected from D*_V_* (*V* ranged from 0 to 5 cc, per 0.5 cc) and V*_D_* (*D* ranged from 40 to 75 Gy, per 1 Gy) for a simple comparison.

## Results

### Analysis of Dose Distribution for Symmetrical Segmentation

**Figure 1:**
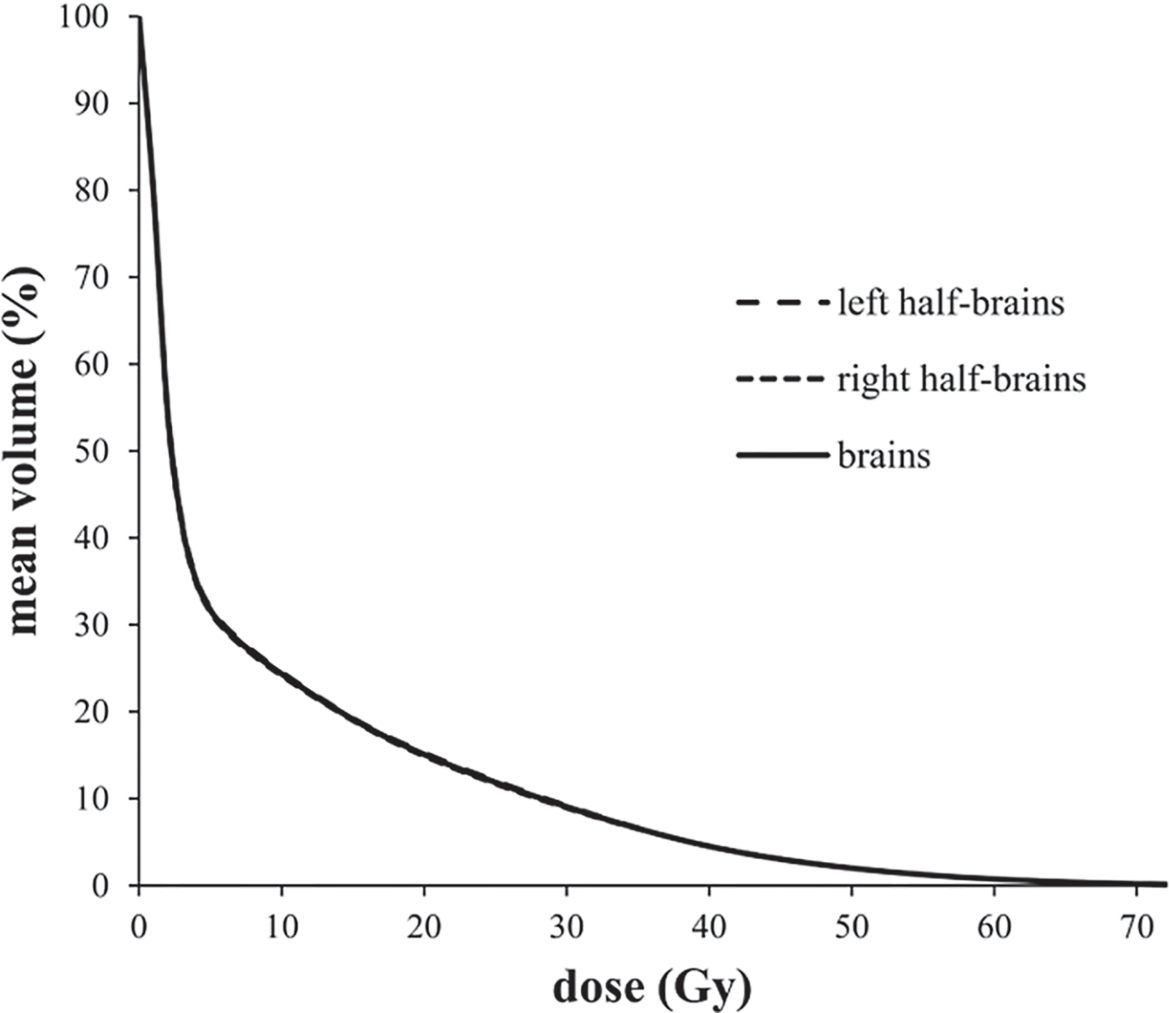
Average-DVH curves of left half-brains, right-half-brains and brains.

Average-DVH curves of left half-brains, right half-brains and brains almost exactly overlapped (**Figure 1**). The V*_D_* proportions of reference volume points (V_10 Gy_, V_20 Gy_, V_30 Gy_, V_40 Gy_, V_50 Gy_, V_60 Gy_, and V_70 Gy)_ of half-brain and whole-brain were enrolled for independent sample *t*-tests. All mean differences of V*_D_* proportions between half-brain and whole-brain were less than 0.01% (P >0.5 in each pair).

### Changes of *a* Value and mAUC in Different Volumes

**Figure 2:**
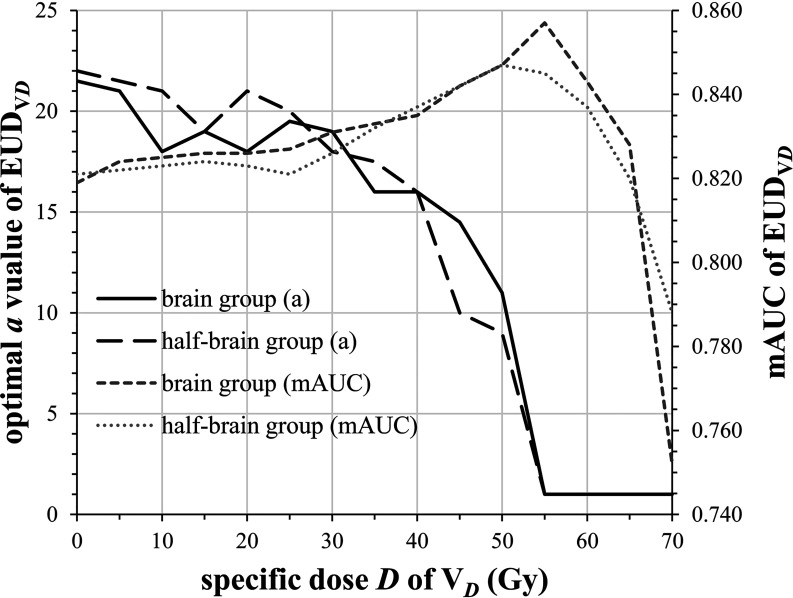
Changes of optimal *a* value and mAUC of EUD_V_ *_D_* as the specific dose *D* changed.

The mAUC of brain EUD was 0.819 and the optimal *a* value was 21.5. Similarly, the mAUC of half-brain EUD was 0.821 and the optimal *a* value was 22. As the specific dose *D* of V*_D_* increased from 0 to 70 Gy (per 5 Gy), the optimal *a* value of brain/half-brain EUD_V_ *_D_* decreased and was always 1 when *D* ≥55 Gy, and the mAUC increased and reached the maximum value when *D* was 50–55 Gy (**Figure 2**). In this study, 55 Gy was selected as a critical dose of EUDVD model for BI.

### Impact of Different Reference Volumes on mAUC and *a* Value

#### Volumes With Similar Dose Distributions But Different Sizes

The mAUCs of EUD_V_ *_D_* (*D* = 0–70 Gy, per 5 Gy) in brain group and half-brain group were enrolled for paired samples *t*-tests. Again, the optimal *a* values of EUD_V_ *_D_* in brain group and half-brain group were enrolled for paired sample *t*-tests. The differences of both AUCs and *a* values between brain group and half-brain group were not significant (P = 0.869/0.834).

#### Volume With Different Dose Distribution (*D* ≤55 Gy)

When *D* was less than or equal to 55 Gy, mAUC and optimal *a* value of EUD_V_ *_D_* were correlated with *D* strongly (**Table 2**). In the range of *a* value from 1 to 22, AUC of brain EUD_V55 Gy_ (0.830–0.857) was always larger than that of whole brain EUD (0.681–0.819), and similarly, AUC of half-brain EUD_V55 Gy_ (0.830–0.845) was always larger than that of whole half-brain EUD (0.691–0.821) (paired sample *t*-tests, P <0.001).

**Table 2:** Pearson correlation coefficients between *D*, mAUC, and *a* in EUD_V_ *_D_* (P <0.01, 2-tailed).

	*D*	mAUC (brain)	*a* (brain)	mAUC (half-brain)	*a* (half-brain)
*D*	1	0.932	−0.813	0.912	−0.877
mAUC (brain)	0.932	1	−0.947	0.937	−0.984
*a* (brain)	−0.813	−0.947	1	−0.829	0.952
mAUC (half-brain)	0.912	0.937	−0.829	1	−0.915
*a* (half-brain)	−0.877	−0.984	0.952	−0.915	1

D, specific dose of V_D_; mAUC, maximal AUC; a, volume-effect parameter of EUD_V D_; EUD_V D_, EUD of V_D_.

### Comparison of Dosimetric Parameter

The optimal dose/volume points for predicting BI in brain group were D_2.5 cc_ and V_70 Gy_, and those in half-brain group were D_1 cc_ and V_69 Gy_. For a simple comparison, AUCs, cutoffs, and Youden indices of optimal dosimetric parameters were calculated and listed (**Table 3**).

**Table 3:** Comparison of dosimetric parameters for brain injury prediction.

	AUC (95% CI)	Cutoff	Sensitivity	Specificity	Youden index
brain					
EUD_V55 Gy_	0.857 (0.775–0.938)	61.80 Gy	0.758	0.857	0.615
D_2.5 cc_	0.801 (0.708–0.895)	67.54 Gy	0.697	0.829	0.526
V_70 Gy_	0.823 (0.735–0.912)	1.37 cc	0.758	0.829	0.586
half-brain					
EUD_V55 Gy_	0.845 (0.776–0.914)	61.31 Gy	0.786	0.811	0.597
D_1 cc_	0.818 (0.748–0.888)	67.22 Gy	0.786	0.756	0.542
V_69 Gy_	0.827 (0.759–0.896)	0.62 cc	0.786	0.756	0.542

### Feasible Volume of Mean Dose

**Figure 3:**
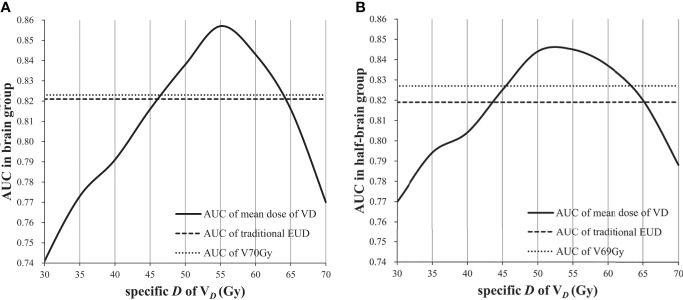
AUC of mean dose of V*_D_* as *D* changed: **(A)** AUC in brain group; **(B)** AUC in half-brain group.

The best volume-effect parameter might be 1 when reference volume was covered by doses not less than critical dose. When *a* value is equal to 1, EUD is equal to mean dose. Therefore, the feasible volume of mean dose was analyzed. The AUC of mean dose of V*_D_* was not inferior to that of traditional EUD when *D* was 45–65 Gy, and it was not inferior to that of dose/volume points when *D* was 50–60 Gy (**Figure 3**).

## Discussion

Modern radiotherapy techniques such as IMRT, volumetric arc radiotherapy (VMAT), and stereotactic body radiotherapy (SBRT) are able to spare normal tissue and reduce side effects (5), resulting in rather non-uniform dose distributions for normal tissue (14). To make sure the traditional NTCP models based on uniform dose still work, effective volume reduction scheme and effective dose reduction scheme have been proposed (15–17). Together, the EUD equation and the Lyman assumptions are often referred to as the Lyman–Kutcher–Burman (LKB) model which has been widely used (5). Other EUD-related models include Logit-EUD model, Schultheiss model (18), Poisson-EUD model, Källman model (19), and Parallel model (20). Regardless of which model is selected to fit clinical data, the intrinsic difference of dichotomous data is the basis of prediction accuracy. In the study Ospina, the six NTCP models above showed almost identical predictive ability for late bladder complications with similar ROC (21). EUD is essentially a redistribution function of dichotomous data. To improve its predictive power, EUD must be able to improve the AUC.

EUD-based optimization of radiation plan has been reported in some studies (22–24). However, there is no guarantee that the parameters are generic and stable in different radiotherapy techniques (such as IMRT and SBRT). As mentioned in the introduction, the volume-effect parameter might be influenced by the dose distribution. In this study, radiation-induced BI model for NPC patients was selected as the object due to its following advantages: 1) fewer setup errors and organ movements; 2) clear boundaries for delineation; 3) clear imaging findings; 4) clear dose–injury relationship with almost no effects of infection or other factors; 5) symmetrical structure and dose distribution for symmetrical segmentation; and 6) relatively concentrated high-dose region and relatively independent injury in temporal pole for asymmetric segmentation. In this study, average-DVH curves of left half-brain, right half-brain and brain overlapped almost exactly, and the differences were negligible, indicating that half-brain could be viewed as an independent structure with a dose distribution similar to that of brain. In addition, V*_D_* with different *D* could be viewed as reference volumes with different dose distributions.

The purpose of half-brain delineation was to study whether the *a* value and mAUC were influenced by volume size. In this study, both the optimal *a* values and their changes were similar in brain group and half-brain group, and also mAUC, indicating that volume size may not be a significant factor. Instead, the dose distribution may be an important factor. The results showed that the optimal *a* value of EUD_V_ *_D_* was correlated with *D* strongly, and it gradually decreased to 1 with the increase of *D*, indicating that the optimal *a* value is variable if the dose distribution is uncertain. The variation of volume-effect parameter in different volumes may be due to the variation of dose distribution rather than to the variation of volume size itself.

An important role of EUD is to convert the doses of entire organ to suit the findings of Emami (25). However, modern radiotherapy techniques rarely involve one third, one half or entire organ being irradiated uniformly. The EUD conversion of entire organ doses may reduce the predictive efficacy, especially in a large organ with only a small volume exposed to high dose, since the low-dose volume may affect the role of crucial doses and increase the uncertainty of *a* value. The study of Heemsbergen showed that the local dose–effects were most pronounced in intermediate-high dose regions for late gastrointestinal toxicity (26). If the EUD was limited to a defined high-dose region, the *a* value would be more stable and the AUC should be larger. However, if the volume of critical dose was removed excessively, the AUC would decrease. In this study, the mAUC of EUD_V_ *_D_* reached the maximum value when *D* was around 50–55 Gy, indicating that 50–55 Gy might be the critical low dose for BI. On the other hand, regardless of what *a* value was selected, AUC of EUD_V55 Gy_ was always larger than that of traditional EUD, further supporting the removal of low-dose volume.

It looked like the brain tissue went from being a series organ to being a parallel organ when *a* value decreased from 21.5/22 to 1, which might not fit the original definition of volume-effect parameter. However, volume-effect parameter of series/parallel organ is essentially an indirect estimation for complication which depends on the location and extent of direct biological damage. In this study, volume-effect parameter of whole volume seemed to only play a role in reducing the interference of low-dose volume. If a novel radiotherapy technique leads to more/less low-dose volume, a larger/smaller *a* value may be needed. Therefore, a parallel/series organ should not be defined directly by the volume-effect parameter.

In this study, whole-volume EUD was not superior to all simple dose/volume points, while EUD_V55 Gy_ could improve the predictive ability, although the difference may not be significant (with AUCs within the 95% CI of the others). However, an overwhelming predictor is almost impossible considering that the continuous variables are highly correlated (for example, D_1 cc_ is never significantly superior to D_1.01 cc_), so the significant trend of AUC may also be important. Given a larger AUC and a more stable *a* value, EUD_V_ *_D_* with an optimal *D* should be a better choice which is less influenced by dose distribution. From the results, in order to fit the low-dose volume together, the best weight relationship of critical doses lost, which may be the reason why whole-volume EUD did not show an advantage.

Mean dose may be the case in which EUD_V_ *_D_* has the best predictive ability, but the critical dose needs to be determined first. In this study, the optimal reference volume of EUD model for BI may be V_50 Gy_–V_55 Gy_ (V_46 Gy_–V_53 Gy_ for equivalent dose in 2 Gy per fraction). When the reference volume ranged from V_45 Gy_ to V_65 Gy_, the AUC of mean dose was not inferior to traditional EUD, and when the reference volume ranged from V_50 Gy_ to V_60 Gy_, the AUC of mean dose was better than traditional EUD and dose/volume points, indicating that even if there is some error in critical dose, the predictive ability is still advantageous in a certain dose range. In fact, it is easier and more reliable to find an optimal mean dose than to fit an unstable parameter. On the other hand, since the volume covered by 55 Gy is related to BI and contributes to the predictive ability, the sub-high dose volumes of brain should be fully delineated (not just temporal lobe) and enrolled in optimization plan to better protect brain tissue. EUD_V55 Gy_ may be a suitable dose constraint index for brain.

It should be clarified that the fundamental purpose of different reference volumes is not to study a sub-structure EUD against traditional EUD, but to enlarge the potential errors (EUD with a large low-dose volume is not better than simple dose/volume points; volume-effect parameter is variable in different dose distribution) in a typical organ and try to improve them. In addition, the higher incidence of BI may be related to advanced T-stage and follow-up bias (symptomatic patients were more likely to complete follow-up). There are several limitations in this study. Firstly, the optimal *a* value was selected by simple calculation with limited accuracy, although the optimal EUD_V_ *_D_* does not need an “accurate” *a* value other than 1. Secondly, the optimal *a* value of 1 is just an assumption that requires more evidence. Thirdly, the conclusions may not be applicable to other organs and more researches are needed. Finally, the division of reference volume is crude, and further studies and more accurate control groups are needed.

## Conclusion

Volume-effect parameter of EUD is variable and related to dose distribution. Before referencing a EUD-based model, the similarity of dose distribution should be confirmed. EUD with large low-dose volume may not be better than simple dose/volume points. EUD of critical-dose volume could improve the predictive ability and has an invariant volume-effect parameter. No matter what *a* value is selected, critical-dose-volume EUD may always be better than whole-volume EUD. Mean dose may be the case in which critical-dose-volume EUD has the best predictive ability.

## Data Availability Statement

The raw data supporting the conclusions of this article will be made available by the authors, without undue reservation.

## Ethics Statement

The studies involving human participants were reviewed and approved by the Ethics committee of Second Affiliated Hospital of Guangxi Medical University. The patients/participants provided their written informed consent to participate in this study.

## Author Contributions

W-QL and R-SW participated in the study concept and design. H-JZ, H-YY, X-DL, XO, Q-LZ, D-JL, Y-TX, and Q-FL participated in the acquisition of data. Q-HD and JL participated in the analysis and interpretation of data. Q-HD participated in the statistical analysis. Q-HD and Y-XG participated in the drafting of the manuscript. All authors contributed to the article and approved the submitted version.

## Funding

This work was supported by the Science Foundation of Guangxi Zhuang Autonomous Region Health and Family Planning Commission [award Z20181010] and the Science Foundation of Second Affiliated Hospital of Guangxi Medical University [award EFYKY2020008].

## Conflict of Interest

The authors declare that the research was conducted in the absence of any commercial or financial relationships that could be construed as a potential conflict of interest.

## Publisher’s Note

All claims expressed in this article are solely those of the authors and do not necessarily represent those of their affiliated organizations, or those of the publisher, the editors and the reviewers. Any product that may be evaluated in this article, or claim that may be made by its manufacturer, is not guaranteed or endorsed by the publisher.
